# A Role for Non‐Canonical Caspases in Fungal Allergic Airway Disease

**DOI:** 10.1111/cea.70237

**Published:** 2026-02-12

**Authors:** Thomas J. Williams, Nazanin Zounemat Kermani, Luis E. Gonzales‐Huerta, Anand Shah, Ian M. Adcock, Kian Fan Chung, Darius Armstrong‐James, U‐BIORPED Study Group

**Affiliations:** ^1^ Department of Infectious Diseases, Faculty of Medicine Imperial College London London UK; ^2^ Data Science Institute Imperial College London London UK; ^3^ National Heart and Lung Institute Imperial College London London UK; ^4^ Department of Respiratory Medicine Royal Brompton and Harefield Hospitals, Guy's and St Thomas' NHS Foundation Trust London UK

## Abstract

- Inhibition of the murine ortholog caspase‐11 reduces neutrophilia and inflammatory cytokine levels.
- Wedelolactone or its derivatives offer a potential therapeutic approach for mixed or steroid‐resistant inflammation in allergic fungal airway disease.

To the Editor,

*Aspergillus fumigatus* is a saprophytic filamentous fungus found ubiquitously in the air we breathe and, although generally innocuous, exposure drives disease in populations such as asthmatics [1]. *Aspergillus* exposure in asthmatics can lead to severe asthma with fungal sensitisation (SAFS), estimated to impact 6–15 million asthmatics, or the more severe allergic bronchopulmonary aspergillosis (ABPA) which has an incidence rate of 2.5% in asthmatics [2, 3]. Long‐term ABPA is associated with recurrent asthma exacerbations, bronchiectasis development, irreversible lung damage and the development of invasive disease and it is characterised by cutaneous reactivity to *A. fumigatus* , elevated IgE serum levels and peripheral blood eosinophilia [4].

Increased caspase‐4 expression in alveolar macrophages has previously been shown in asthmatics compared with healthy controls. The same study showed that the murine ortholog caspase‐11, the driver of non‐canonical inflammasome activation, is implicated in the generation of allergic asthma and facilitates the release of proinflammatory IL‐1 cytokines [5]. To investigate whether there are direct contributions of non‐canonical caspases in the generation of fungal allergic airway disease we utilised a murine repeat challenge model (Figure 1A). Wedelolactone, a coumestan compound extracted from *Wedelia calendulacea* or *Eclipta alba* , reported to inhibit caspase‐11 activity and downstream inflammasome signalling, was employed as a pharmacological tool to probe the contribution of caspase‐11‐associated pathways in fungal allergic airway inflammation.

**FIGURE 1:**
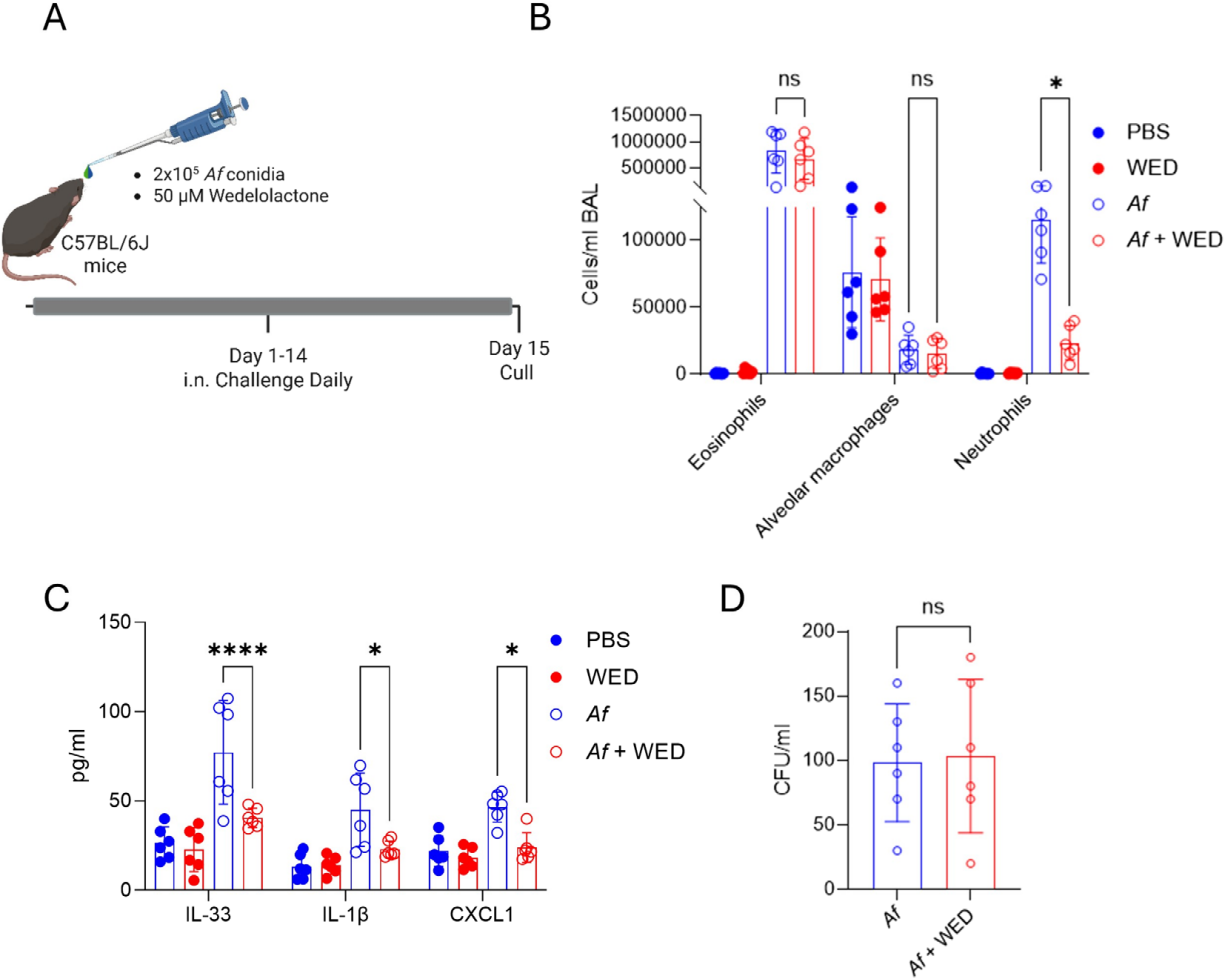
Wedelolactone reduces neutrophilia in fungal allergic airway disease without impairing fungal control. (A) Mice were dosed daily with or without 2 × 10^5^ live *A. fumigatus* (*Af*) conidia and treated with or without wedelolactone (WED) (50 μm) for 14 days and culled 24 h after the final dose. Bronchoalveolar lavage fluid (BALF) was collected and assessed for (B) eosinophil, alveolar macrophage and neutrophil counts by flow cytometry as well as (C) IL‐33, IL‐1β and CXCL1 by ELISA, and (D) CFU counts. (*n* = 6 per group, from two independent experiments). Data are represented as the mean ± SEM. (B–D) Students *t*‐test, *p < 0.05, ****p < 0.0001, ns—not significant.

In this model mice were dosed intranasally with 50 μL of PBS or 2 × 10^5^ live *A. fumigatus* conidia with wedelolactone (50 μM) or vehicle control for 14 days. Daily intranasal wedelolactone was selected to coincide with each low‐dose intranasal *Aspergillus* conidial challenge, thereby ensuring drug availability at the airway mucosa during the repeated allergen exposures that drive sensitisation and inflammation. This concentration was chosen based on prior reports that wedelolactone exerts anti‐inflammatory effects in vitro and in vivo at micromolar levels without overt cytotoxicity, and on its relatively short systemic half‐life and limited bioavailability, which together support the use of a locally delivered, pharmacologically active dose to achieve sufficient mucosal exposure over the sensitisation period. Intranasal administration was used to deliver wedelolactone directly to the nasal and upper airway surfaces, the primary sites of conidial deposition and immune activation, allowing high local concentrations while minimising systemic exposure and potential off‐target effects. No mortality or significant weight loss was observed during the study (data not shown). Twenty‐four hours after the final dosing, the mice were culled and bronchoalveolar lavage (BAL) was performed.

Flow cytometry was used to investigate how inhibition of caspase‐11 impacted infiltration of immune cells into the airway. There was no reduction in eosinophilia or rescue of alveolar macrophages with wedelolactone treatment of *A. fumigatus* exposed mice; however, there was a significant reduction in the number of neutrophils present when compared with untreated infected controls (Figure 1B). In addition, reductions of IL‐33, IL‐1β and CXCL1 levels in the BAL fluid were observed in mice treated with wedelolactone when compared with untreated controls (Figure 1C). This reduction in neutrophil influx and inflammatory cytokine production did not increase the fungal burden of the lung (Figure 1D). These data suggest that while inhibition of caspase‐11 does not alter eosinophil influx, caspase‐11 is required for neutrophil recruitment during the allergic response and its blockade suppresses the levels of inflammatory cytokines.

Previous studies have shown a similar reduction of neutrophil influx and IL‐1β release in an *Aspergillus* keratitis model treated with wedelolactone, corroborated by caspase‐11 knockouts which show the same phenotype, highlighting that neutrophil caspase‐11 in particular is required for IL‐1β secretion in *A. fumigatus* infection [6, 7]. A previous study of house dust mite (HDM) allergy has shown that IL‐4 and IL‐13 promote caspase‐11 expression and that during HDM allergy in caspase‐11 knockouts there is a reduction in neutrophilia and the production of proinflammatory cytokines including IL‐1β, but a significant increase in eosinophilia [8]. In contrast, caspase‐11 knockouts undergoing an ovalbumin model of allergy showed a significant reduction of eosinophilia [5]. In our live *Aspergillus* repeat‐challenge model, caspase‐11 inhibition reduced neutrophilia but had no impact on eosinophilia, likely due to the fact that live conidia drive mixed inflammation with a prominent neutrophil‐dominant component via caspase‐11‐sensitive pathways (e.g., IL‐17/IL‐23, CXCL chemokines, non‐canonical inflammasome activation) alongside a Th2/eosinophil axis reliant on downstream cytokines (IL‐5/IL‐13) that operate independently from caspase‐1/NLRP3 signalling. The discordant changes in airway cells between these models is likely due to the employment of live pathogen compared with ovalbumin or HDM, which may have different binding profiles for caspase‐11 activation and preferentially amplify early neutrophil responses over adaptive eosinophil recruitment. These data partially corroborate the shift in the neutrophilic allergic phenotype seen in this work, however the model employed in this work delivered caspase‐11 inhibitor directly to the lung rather than utilising global knockout or systemic inhibition, suggesting that local versus systemic caspase‐11 inhibition may exert site‐specific effects on immune cell recruitment. Importantly, in our study wedelolactone treatment reduced inflammatory cell recruitment and cytokine production without increasing fungal burden, however extended time‐course studies will be required to determine whether wedelolactone alters fungal clearance kinetics or promotes delayed pathogen persistence.

While wedelolactone has been reported to inhibit caspase‐11 activity and downstream non‐canonical inflammasome signalling, it also modulates additional pathways, including NF‐κB activation, inducible nitric oxide synthase (iNOS) expression and cyclooxygenase (COX) activity [9, 10]. Therefore, we cannot exclude the possibility that some of the observed anti‐inflammatory effects arise from off‐target interactions or broader suppression of inflammatory signalling. Nevertheless, the observed reduction in neutrophil recruitment and IL‐1β release is consistent with phenotypes reported in caspase‐11‐deficient models of fungal infection, supporting the conclusion that caspase‐11‐dependent mechanisms likely contribute to the responses observed here. Future studies utilising this model with knockout animals or more selective inhibitors will be essential to definitively confirm the role of caspase‐11 in fungal allergic airway inflammation.

As ABPA and SAFS are typically dominated by eosinophilic inflammation, our initial goal was to dampen this response through caspase‐11 pathway modulation using wedelolactone. Unexpectedly, however, wedelolactone selectively reduced neutrophilic inflammation while leaving eosinophilia unchanged. Neutrophil‐driven responses play a key role in disease severity and steroid resistance, and that severe or steroid‐refractory asthma often presents with mixed eosinophilic and neutrophilic inflammation [11]. Thus, targeting caspase‐11‐linked neutrophilic pathways may offer a complementary strategy to current anti‐eosinophil therapies, such as anti‐IL‐5 biologics, by mitigating the persistent neutrophilic component that contributes to ongoing airway inflammation and poor clinical control.

Although current therapeutics for caspase targeting are limited due to high toxicity and poor bioavailability, the increasing evidence for a role of caspase‐4 in multiple inflammatory diseases is driving the development of novel inhibitors [12]. The results shown here indicate the potential for targeting these pathways for the treatment of inflammation in fungal allergic airway disease and potentially responses to other aeroallergens.

## Author Contributions

T.J.W., L.E.G.‐H., A.S. and D.A.‐J.: conceptualised the study; T.J.W. and L.E.G.‐H.: were associated with methodology; T.J.W. and N.Z.K.: performed formal analysis; T.J.W, L.E.G.‐H.: and N.Z.K.: performed the investigations; T.J.W.: wrote the original draft; all authors were associated with review and editing of the final manuscript; T.J.W.: visualised the study; A.S., I.A., K.F.C.: and D.A.‐J.: supervised the study; A.S.: and D.A.‐J.: acquired funding for the study.

## Funding

T.J.W. and D.A.‐J. were supported by funding from the Cystic Fibrosis Trust (grant no. SRC015 and THUB02).

## Conflicts of Interest

The authors declare no conflicts of interest.

## Data Availability

All data supporting the findings of this study are contained in the text and figures.
